# Evidence for serial founder events during the colonization of North America by the yellow fever mosquito, *Aedes aegypti*

**DOI:** 10.1002/ece3.8896

**Published:** 2022-05-13

**Authors:** Evlyn Pless, Jeffrey R. Powell, Krystal R. Seger, Brett Ellis, Andrea Gloria‐Soria

**Affiliations:** ^1^ 5758 Department of Ecology and Evolutionary Biology Yale University New Haven Connecticut USA; ^2^ Department of Anthropology University of California Davis California USA; ^3^ U.S. Virgin Islands Department of Health Christiansted VI USA; ^4^ Department of Environmental Sciences The Connecticut Agricultural Experiment Station New Haven Connecticut USA

**Keywords:** *Aedes aegypti*, genetic structure, Invasive species, North America, population genetics, serial founder effect

## Abstract

The *Aedes aegypti* mosquito first invaded the Americas about 500 years ago and today is a widely distributed invasive species and the primary vector for viruses causing dengue, chikungunya, Zika, and yellow fever. Here, we test the hypothesis that the North American colonization by *Ae*. *aegypti* occurred via a series of founder events. We present findings on genetic diversity, structure, and demographic history using data from 70 *Ae*. *aegypti* populations in North America that were genotyped at 12 microsatellite loci and/or ~20,000 single nucleotide polymorphisms, the largest genetic study of the region to date. We find evidence consistent with colonization driven by serial founder effect (SFE), with Florida as the putative source for a series of westward invasions. This scenario was supported by (1) a decrease in the genetic diversity of *Ae*. *aegypti* populations moving west, (2) a correlation between pairwise genetic and geographic distances, and (3) demographic analysis based on allele frequencies. A few *Ae*. *aegypti* populations on the west coast do not follow the general trend, likely due to a recent and distinct invasion history. We argue that SFE provides a helpful albeit simplified model for the movement of *Ae*. *aegypti* across North America, with outlier populations warranting further investigation.

## Graphical abstract

We find support for the hypothesis that the North American colonization by the yellow fever mosquito (*Aedes aegypti*) occurred via a series of founder events with Florida as the putative source. We present findings on genetic diversity, structure, and demographic history using data from 70 *Ae*. *aegypti* populations in North America that were genotyped at 12 microsatellite loci and/or ~20,000 single nucleotide polymorphisms, the largest genetic study of the region to date.

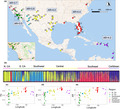

## 1 INTRODUCTION

Range expansion is the process by which invasive species spread to new regions and environments. This process can be modeled as a continuous expanding wave, a series of founder events, or stochastic jumps (Peischl et al., 2016). Each type of range expansion leads to characteristic genetic patterns that are also influenced by genetic drift and gene flow (Excoffier et al., 2009). Understanding the dynamics of range expansion for a given invasive species can shed light on their demographic history and may illuminate options for preventing future spread.

The *Aedes aegypti* mosquito (Linnaeus, 1762) is an invasive species that has successfully invaded tropical regions around the world and is increasingly reported in temperate regions (Kraemer et al., 2015), with a rate of invasion that is expected to accelerate with climate change (Iwamura et al., 2020). The global distribution of this vector enabled the recent outbreaks of Zika and chikungunya in the Americas (Carlson et al., 2016; Leparc‐Goffart et al., 2014), reemergence of yellow fever in Africa and South America (Hamlet et al., 2018), and a dramatic spread and increase of dengue cases around the world (Brady & Hay, 2020). Its widespread presence in Mexico and the Caribbean perpetuates endemic dengue, and its presence in the southern United States poses a public health threat.

*Aedes aegypti* likely arrived in the Americas in the 17th century aboard slave ships from Africa, where it rapidly spread throughout the continent, as evidenced by outbreaks of yellow fever and dengue; see the comprehensive review (Powell et al., 2018). Disease outbreaks ranged from the Caribbean to the North Atlantic and the southeast United States by the early 1800s (Carrigan, 1959; Moreno‐Madriñán & Turell, 2018). Dengue made its way into the central‐south United States in the 1850s (Chandler, 1956), and by the 1930s *Ae*. *aegypti* spanned much of Texas and coastal Mexico (Slosek, 1986). *Ae*. *aegypti* became more prevalent and widespread in New Mexico around 1994 (Merrill et al., 2005) and in California starting around 2013 (Metzger et al., 2017). Taken together, these lines of evidence paint a picture of the westward migration of the mosquito species.

When a small number of founders emigrate from a larger population, the new population will generally display a reduction in genetic variation relative to the original population that can last for many generations—a phenomenon called “founder effect” (Nei et al., 1975). The serial founder effect (SFE) (spread occurring through a series of these founder events) has been famously invoked to describe the movement of *Homo sapiens* out of Africa (Henn et al., 2012; Ramachandran et al., 2005). It has also been used to explain the global spread of the malaria parasite that accompanied humans out of Africa and the expansion of monarch butterflies across the Pacific (Pierce et al., 2014). Although numerous studies have examined *Ae*. *aegypti* invasions, migration, and structure using populations genetics from around the globe—including North America (Gloria‐Soria et al., 2016; Kotsakiozi et al., 2018; Pless et al., 2017)—none have explicitly tested the hypothesis of colonization by SFE.

Here, we asked whether SFE explains the westward spread of *Ae*. *aegypti* across southern North America. These migrations were likely accomplished by a combination of active dispersal and passive human‐mediated transport (e.g., trucks and ships) (Fonzi et al., 2015; Goncalves da Silva et al., 2012; Guagliardo et al., 2014; Medley et al., 2015). Given an active average lifetime dispersal of <200 m for *Ae*. *aegypti* (Honorio et al., 2003; Jasper et al., 2019; Reiter, 2007; Russell et al., 2005), we predict that short‐distance invasions are more common for both active and passive dispersal due to a higher number of introduction events and higher propagule pressure (Sakai et al., 2001), which is likely to result in colonization via SFE.

To test if *Ae*. *aegypti* expansion across North America is consistent with a SFE moving westward, we evaluated our data against four expectations:

1. Source populations will have the highest number of alleles, with genetic diversity decreasing in proportion to the distance from the source (Hunley et al., 2012).
2. There will be a positive relationship between distance among groups and their genetic differentiation (Ramachandran et al., 2005).
3. Following each founding event, the daughter group will carry a subset of variation from the parental group (Ramachandran et al., 2005).
4. Demographic inference modeling will support SFE over other possible scenarios.

We inferred genetic structure across the region and tested these predictions using data from 12 microsatellite loci and single nucleotide polymorphism (SNP) array data from 70 North American *Ae*. *aegypti* populations. Establishing the extent to which *Ae*. *aegypti* has spread via SFE is important for preventing and detecting future invasions, as well as modifying vector control in response to the movement of pesticide‐resistant alleles.

## 2 MATERIALS AND METHODS

### 2.1 Mosquito collection

**FIGURE 1:**
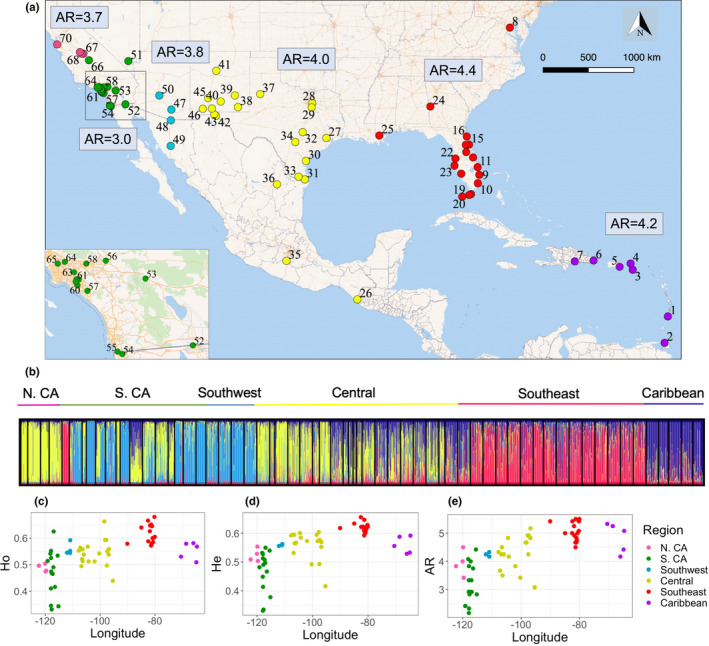
(a) Map showing locations for each *Aedes aegypti* sampling site included in this study, colored by regional group. The inset on the bottom left shows southern California. Mean allelic richness estimated from microsatellites by rarefaction of all sites within each region is shown in the boxes. (b) STRUCTURE plot of the complete microsatellite dataset with K = 4 number of clusters. Each column is an individual, and the heights of the color bars represent the proportion of ancestry that came from each of the four inferred clusters (yellow, light blue, red, and dark blue). The outlier (red) in southern California is Exeter County. The longitude of each site is plotted against its mean observed heterozygosity (c), expected heterozygosity (d), and allelic richness estimated by rarefaction using microsatellite data (e). Colors in the map (a), above the STRUCTURE plot (b), and in the diversity plots (c–e) are consistent and correspond to regional groups

Our analysis includes 70 *Ae*. *aegypti* populations across continental North America and the eastern Caribbean (Figure 1, Table 1 and Table S1). Microsatellite and SNP genotypes for most populations in this study have been reported in Evans et al. (2015), Gloria‐Soria et al. (2014), Gloria‐Soria et al. (2016), Kotsakiozi et al. (2017), Pless et al. (2017), Pless et al. (2020), Pless et al. (2021) and Saarman et al. (2017). New data presented here include microsatellite genotypes from (1) La Altagracia, Dominican Republic, (2) San Jose de Ocoa, Dominican Republic, (3) St. Croix, USVI, and (4) St. Thomas, USVI, and genome‐wide SNP data for (1) St. Thomas, USVI, (2) Alamagordo, NM, USA, (3) Las Cruces, NM, USA, (4) Lubbock, TX, USA, and (5) Bexar, TX, USA. The new populations genotyped fill important regional gaps, particularly in the Caribbean and the central United States. The remaining gaps in samplings, such as those between the panhandle of Florida and New Orleans, are due to the absence of *Ae*. *aegypti* in recent years.

**TABLE 1:** *Aedes aegypti* populations included in this study

Pop. number	Population	Abbrev.	Region name	Year	N^1^	N^2^
1	St. Vincent	SV	Caribbean	2015	0	12
2	Trinidad	TRI	Caribbean	2014	0	12
3*	St. Croix, USVI	StX	Caribbean	2017	32	0
4*^+^	St. Thomas, USVI	StT	Caribbean	2017	36	10
5	Patillas, Puerto Rico	PR	Caribbean	2014	40	12
6*	La Altagracia, DR	Alt	Caribbean	2018	31	0
7*	San Jose de Ocoa, DR	Oco	Caribbean	2018	39	0
8	Washington, D.C.	WashDC	Southeast	2014	0	11
9	Palm Beach County, FL, USA	PBC	Southeast	2013	40	12
10	Miami, FL, USA	Miami	Southeast	2011	40	8
11	Rio, FL, USA	FLO	Southeast	2014	40	0
12	Melbourne, FL, USA	Mel	Southeast	2014	40	12
13	Conch Key, FL, USA	Conch	Southeast	2006	40	0
14	Vaca Key, FL, USA	Vaca	Southeast	2009	40	0
15	Daytona Beach, FL, USA	DB	Southeast	2017	40	12
16	St. Augustine, FL, USA	StA	Southeast	2017	40	0
17	Orlando, FL, USA	Orl	Southeast	2014	32	11
18	Barberville, FL, USA	Bb	Southeast	2017	40	11
19	North Key West, FL	NK	Southeast	2013	0	11
20	Key West, FL, USA	KW16	Southeast	2016	40	12
21	Fort Myers, FL, USA	FM	Southeast	2014	37	12
22	Tampa, FL, USA	Tam	Southeast	2014	40	12
23	Sarasota, FL, USA	Sar	Southeast	2014	39	12
24	Muscogee, GA, USA	18 and 9	Southeast	2011	40	0
25	New Orleans LA, USA	NO and NO2	Southeast	2012	40	10
26	Tapachula Norte, CHP, MEX	TapaN	Central	2012	0	12
27	Houston, TX, USA	Houston and H11	Central	2011	19	8
28	Dallas, TX, USA	Dall	Central	2017	40	0
29	Ellis, TX, USA	El	Central	2017	40	0
30	Nueces, TX, USA	Nuec	Central	2017	40	0
31	Cameron, TX, USA	Cam	Central	2015	40	0
32	Travis, TX, USA	Tr	Central	2017	40	0
33	Hidalgo, TX, USA	Hid	Central	2017	40	0
34^+^	Bexar, TX, USA	Bex	Central	2017	18	8
35	Amacuzac, Morelos, Mexico	Amac16_P	Central	2014	0	12
36	Nuevo Leon, MEX	NL	Central	2017	10	0
37^+^	Lubbock, TX, USA	Lub	Central	2017	14	8
38	Carlsbad, NM, USA	Car	Central	2017	17	0
39	Roswell, NM, USA	Ros	Central	2017	39	0
40^+^	Alamagordo, NM, USA	Ala	Central	2017	35	4
41	Albuquerque, NM, USA	Alb	Central	2018	22	0
42	Juarez, MEX	Juar	Central	2017	40	0
43	Sunland Park, NM, USA	SP	Central	2017	37	0
44^+^	Las Cruces, NM, USA	LC18	Central	2018	40	6
45	Truth of Consequences, NM, USA	TC	Central	2017	17	0
46	Deming, NM, USA	Dem	Central	2017	40	0
47	Tucson, AZ, USA	TJC2	Southwest	2012	40	12
48	Nogales, Son, MEX	Nog	Southwest	2013	40	9
49	Hermosillo, Son, MEX	Her	Southwest	2013	40	0
50	Maricopa County, AZ, USA	Az	Southwest	2013	39	0
51	Las Vegas, NV, USA	LV	Southern CA	2017	31	0
52	El Centro, CA, USA	ElC	Southern CA	2016	40	0
53	Coachella, CA, USA	Coa	Southern CA	2017	27	0
54	Tijuana, BCN, MEX	Tj	Southern CA	2013	20	10
55	San Diego, CA, USA	Cw and SY	Southern CA	2015	40	12
56	San Bernardino, CA, USA	SBern	Southern CA	2017	40	0
57	Mission Viejo, CA, USA	MV	Southern CA	2015	40	12
58	Montclaire, CA, USA	Mc	Southern CA	2016	30	0
59	Orange, CA, USA	Or	Southern CA	2015	13	0
60	Santa Ana, CA, USA	SA17	Southern CA	2017	33	0
61	Anaheim, CA, USA	Ana_LC and Ana	Southern CA	2015	31	0
62	Garden Grove, CA, USA	GG	Southern CA	2015	29	12
63	La Habra, CA, USA	LH	Southern CA	2017	13	0
64	Rosemead, CA, USA	Ro	Southern CA	2017	40	0
65	Los Angeles, CA	GLA	Southern CA	2014	0	6
66	Exeter, CA, USA	Exe	Southern CA	2014	23	12
67	Clovis, CA, USA	Clovis and Cal	Northern CA	2013	40	6
68	Fresno, CA, USA	Fres	Northern CA	2015	27	12
69	Madera, CA, USA	MAD	Northern CA	2015	40	12
70	San Mateo, CA, USA	SM and SM2	Northern CA	2013	22	8

Note

Population number corresponding to Figure 1, population name, population abbreviation, region name, year sampled, the sample size for microsatellite data (N^1^), and sample size for SNP data (N^2^). New microsatellite data are indicated with an asterisk (*) and new SNP data are indicated with a cross (^+^).

Abbreviations: DR, Dominican Republic; MEX, Mexico; USA, United States of America; USVI, United States Virgin Islands.

The mean sample size is 33.8 for microsatellites and 10.4 for SNPs. The year range of sampling collections is 2006–2018 (with >75% of the samples collected in 2014 or later). All mosquito samples were collected as adults or eggs from traps and were shipped as adults to Yale University for analysis. No more than six individuals were used from a single ovitrap to minimize sampling relatives.

### 2.2 DNA extraction and genotyping

The microsatellite dataset includes 2132 individuals from 63 populations genotyped at 12 loci (Brown et al., 2011; Slotman et al., 2007). The SNP dataset includes 373 individuals from 36 populations genotyped at ~20,000 SNPs with the Axiom_aegypti array (Evans et al., 2015). Microsatellites are appropriate for unbiased genetic diversity estimates and demographic inference analysis due to their multiallelic, highly polymorphic nature, and complete allele frequency spectra. Additionally, their low cost facilitates the generation of large global and local reference databases. In contrast, the larger number of markers from the Axiom_aegypti array may provide higher fine‐scale resolution for population structure, particularly when populations are of recent origin or when gene flow is significant (Gloria‐Soria et al., 2018). However, the ascertainment bias in the design of the SNP array is likely to affect measurements of genetic diversity and may also impact demographic analysis, which uses allele diversity spectrum to evaluate the likelihood of different scenarios. Therefore, the two sets of genetic markers provide complementary advantages and are useful for different analyses.

Whole genomic DNA was extracted from individual mosquitoes using the Qiagen DNeasy Blood and Tissue kit according to manufacturer instructions, including the optional RNAse A step. Individuals were genotyped for 12 microsatellites as in Gloria‐Soria et al. (2016). Four loci (A1, B2, B3, and A9) are trinucleotide repeats, and eight (AC2, CT2, AG2, AC4, AC1, AC5, AG1, and AG4) are di‐nucleotide repeats. Any individuals that were genotyped at fewer than 10 loci were excluded from the analysis. We include only populations with a minimum of 10 individuals and arbitrarily selected 40 individuals from sites with more than 40 samples to control for unequal or low sample size (Puechmaille, 2016).

Individuals were genotyped using Axiom_aegypti, a high‐throughput genotyping chip that has 50,000 probes (Evans et al., 2015). Genotyping was conducted by the Functional Genomics Core at the University of North Carolina, Chapel Hill. To prune the SNP dataset, we first excluded 2166 SNPs that failed a test of Mendelian inheritance (Evans et al., 2015). Since some analyses can be confounded by SNPs in linkage disequilibrium (Alexander et al., 2009), we excluded tightly linked SNPs with the plink command “‐‐indep 75 kb 50 2” (Purcell, 2016; Purcell et al., 2007). We also excluded any SNPs that were genotyped in <90% of the individuals and those with a minor allele frequency of <5%, resulting in 20,003 SNPs remaining for analysis.

All microsatellite data are available in Dataset S1, and all SNP data are available in Dataset S2. Additionally, the data can be accessed on Dryad (https://doi.org/10.5061/dryad.5x69p8d5j) and VectorBase (VBP0000801).

### 2.3 Inferring geographic regions based on genetic structure

All microsatellite loci were tested for within‐population deviations from Hardy–Weinberg equilibrium and for linkage disequilibrium among loci pairs using the R package Genepop v. 1.1.4. with 5000 dememorizations, 500 batches, and 5000 iterations per batch for both tests (Raymond & Rousset, 1995). To correct for multiple testing, a Bonferroni correction was applied at the .05 α level of significance.

To establish regional groupings for subsequent analysis, we examined population structure using a number of methods. We performed principal component analysis (PCA) using the R package Adegenet v. 2.1.1. (Jombart, 2008) for the microsatellites. We conducted 20 independent runs of STRUCTURE v. 2.3.4 for K = 1–10 (Pritchard et al., 2000) using the microsatellite data and 600,000 generations, with the first 100,000 discarded as burn‐in. We visualized the STRUCTURE results using the programs Clumpak and DISTRUCT v.1.1 (Kopelman et al., 2015; Rosenberg, 2004), and we inferred the optimal value of K using relevant guidelines (Cullingham et al., 2020; Earl, 2012; Evanno et al., 2005). We repeated these analyses for the Caribbean microsatellite dataset (40 independent runs of STRUCTURE for K = 1–6), and for the Central and Southwest microsatellite datasets combined (20 independent runs of STRUCTURE for K = 1–10) to further explore the regional genetic structure of the two regions that include new populations. Additionally, we evaluated genetic structure within the Caribbean using a multivariate approach, Discriminant Analysis of Principal Components (DAPC), on the microsatellite data using the Adegenet package (Jombart, 2008).

A PCA for the complete SNP dataset was generated with Plink v.1.9 (Purcell, 2016; Purcell et al., 2007). Additionally, we ran 5 independent runs in fastSTRUCTURE 1.0 (Raj et al., 2014) using the SNP dataset for K = 1–10, and visualized the results using Clumpak and DISTRUCT v.1.1 (Kopelman et al., 2015; Rosenberg, 2004).

Based on the results from our genetic clustering analysis, we grouped the samples into six regions for further analyses (Table 1, Figure 1): Caribbean contains the Caribbean; Southeast contains Florida, Louisiana, Georgia, and Washington D.C.; Central contains eastern Texas, western Texas, New Mexico; Southwest contains Arizona; Southern CA contains southern California and Nevada; and Northern CA contains northern/central California.

### 2.4 Prediction 1: Genetic diversity decreases toward the west

Observed heterozygosity (H_O_), expected heterozygosity (H_E_), the inbreeding coefficient (F_IS_), and a number of private alleles (alleles found in no other population) were calculated from the microsatellite dataset for each population using GenAlEx v. 6.51 (Peakall & Smouse, 2006), and allelic richness was estimated by rarefaction (*N* = 30) using the software HP‐Rare v. 1.0 (Kalinowski, 2005). These measurements were not calculated for the SNP dataset due to ascertainment bias in the design of the SNP array (Evans et al., 2015).

To assess if genetic diversity decreased westward, as expected from SFE, we calculated linear regressions for longitude versus the different genetic diversity metrics (observed heterozygosity, expected heterozygosity, and allelic richness) in R v. 4.0.2 (R Core Team, 2020). To determine whether Florida or the Caribbean was the more likely source of the expansion, we also calculated linear regressions for observed heterozygosity, expected heterozygosity, and allelic richness of each population versus (1) their distance to Florida and (2) their distance to the Caribbean.

We assessed regional genetic diversity by averaging the genetic diversity metrics across all the sites within each region. Since regional allelic richness and the number of private alleles could be biased by the number of individuals in a region, we calculated the genetic diversity measures a second time after combing all individuals within each region and then randomly resampling them so that each region had the same number of individuals.

### 2.5 Prediction 2: Positive relationship between geographic and genetic distance

Using both the microsatellite and SNP datasets, we calculated pairwise *F_ST_* and evaluated significance with 1000 permutations using Arlequin 3.5 (Excoffier et al., 2005). We then tested for a relationship between pairwise genetic distance $\left(\frac{F_{\mathrm{ST}}}{\left(1‐F_{\mathrm{ST}}\right)}\right)$ and geographic distance using a Mantel test with 9999 permutations, and repeated the test after excluding known new invasions, defined as populations that were first detected in 2013 or later (California, Las Vegas NV, and Albuquerque NM).

Because clustering and assignment methods may mistake continuous processes (e.g., isolation by distance, in which there is a positive relationship between geographic distance and gene flow between populations) for discrete processes, we implemented a method called conStruct v. 1.0.4 (Bradburd et al., 2018) using the SNP array data. This model‐based clustering method uses isolation by distance when possible to explain genetic variation. We ran three independent runs of the program for K = 1–4 with 1000 iterations and the spatial model setting and another three independent runs with the non‐spatial model setting. We assessed posterior probability, Markov chain Monte Carlo (MCMC) performance, and layer contributions to compare the independent runs and identify a putative optimal number of layers for each run. The program performs best when there are more loci than the number of samples, so it was not suitable to run with the microsatellite data.

### 2.6 Prediction 3: Daughter groups nested within parental group

To test the hypothesis that daughter groups contain a subset of the allelic diversity from parent groups, we created the presence/absence matrices representing all alleles for each of the 12 microsatellites, where regional sites were columns, and the different alleles were rows. Microsatellites were used rather than SNPs because the latter are biallelic, and thus do not have the broad allele frequency spectra required for this type of analysis. To maximize the chance of finding a pattern if one existed, we focused on three regions that were most likely to display this pattern: Southeast, Central, and Southwest. To control for uneven sample size across different populations, we combined individuals within each region and resampled, so each region was represented by the same number of individuals before analysis. We calculated the nestedness metric based on the overlap and decreasing fill (NODF) for each matrix, in which higher scores indicate greater amounts of nestedness (Morrison, 2013), using the R package “RInSp” (Zaccarelli et al., 2013). To compare our matrices and their NODF scores with the null expectation, we created five control model matrices by shuffling the elements in each row using the R package “picante,” specifically the randomizeMatrix tool, with 1000 iterations and the null model set as “richness” (Kembel et al., 2010).

### 2.7 Prediction 4: Demographic inference modeling supports SFE

We performed demographic history analysis using DIYABC‐RF (Random Forest) v.1.1.1‐beta (Collin et al., 2021) on the microsatellite dataset, to avoid possible effects of ascertainment bias on allele frequency spectra derived from the SNP chip selection process. The DIYABC‐RF approach enables efficient discrimination among scenarios and estimation of the posterior probabilities with a lower computational burden than classic approximate Bayesian computation approaches (Collin et al., 2021). The program applies supervised machine learning methods to population genetic data for statistical inference through the use of a training set. The training set includes a given number of datasets simulated under different evolutionary scenarios using parameter values drawn from prior distributions. Priors were set to be as wide as possible within reason given known colonization events (Table S2). Each resulting dataset is then summarized with a set of descriptive statistics. These summary statistics describe genetic variation within populations, between pairs or triplets of populations, averaged over loci. The RF algorithm then chooses the best scenario from the simulated datasets and characterizes the posterior distribution of parameters of interest under a specific scenario and assesses the performance of RF‐based inferences in terms of prediction through the computation of error and accuracy measurements.

**FIGURE 2:**
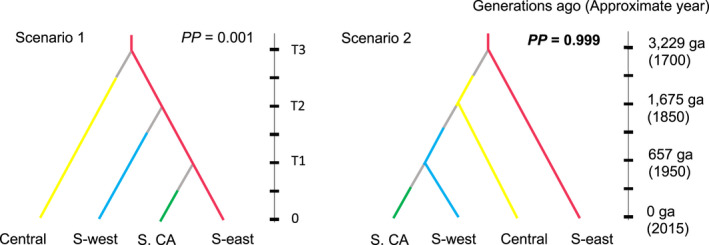
Scenarios tested by DIYABC‐RF using microsatellite data. The two trees depict scenarios of *Ae*. *aegypti* colonization of continental North America. Scenario 1 shows independent invasions from the Southeast with bottlenecks after each lineage division and Scenario 2 shows a pattern of serial founder effect moving west from the Southeast, also with bottlenecks. Posterior probabilities (PP) are shown for each scenario. Time 0 corresponds to the present (when the samples were collected). Colors for each lineage correspond to Figure 1, and changes in effective population size (bottlenecks) are shown in gray. Posterior probabilities and divergence times correspond to a single run of the model using random draws from each region (Table S2b); results are similar for the other three independent runs (Table S2)

Specifically, we compared SFE scenario—in which Southern CA split from Southwest, which split from Central, which split from the Southeast—against a scenario where each region diverged from the Southeast independently (Figure 2). Each region was represented by 158 randomly selected samples, and we performed the analysis in duplicate, using a second random draw from each region. We excluded Exeter from Southern California since it is a known outlier that likely has a different origin than the other sites in Southern California (see Results). To ensure that pooling across populations did not bias the outcome, we also repeated the analysis twice replacing the regions with single, arbitrarily selected populations from each region (Table S2). We ran DIYABC‐RF using a training set of 200,000 simulated datasets. The constructed RF for both model choice and parameter estimation used 1000 trees, and the best scenario was selected based on linear discriminant analysis and partial least squares regression analysis, in each case. Full parameters and priors are listed in Table S2.

## 3 RESULTS

### 3.1 Inferring geographic regions based on genetic structure

We first confirmed that our microsatellites behave as independent, neutral loci by performing 756 Hardy–Weinberg equilibrium (HWE) tests (12 microsatellite loci X 63 populations) and 4158 linkage disequilibrium pairwise tests on loci within populations. After applying a Bonferroni correction (*p *= .05/number of tests), 13 loci‐by‐populations (1.7%) were out of HWE, and there were not enough data to determine the p‐values for 18 tests. Similarly, 57 loci pairs within populations (1.4%) showed significant evidence of being in linkage disequilibrium after a Bonferroni correction, and there were not enough data to determine the p‐values for 127 tests. These observations, 1.7% and 1.4%, are below the “significance” cutoff of 5%.

**FIGURE 3:**
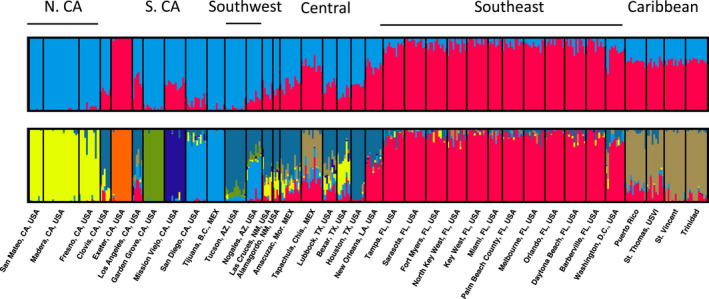
Population structure in North America generated by fastStructure using the SNP dataset. Populations are arranged according to their longitude (west on the left). Each column is an individual, each color represents an inferred group, and the height of the color bar shows the proportion of ancestry that came from the K inferred groups (K = 2 on the top panel and K = 8 on the bottom panel)

To test our hypothesis that *Ae*. *aegypti* primarily colonized North America through a series of westward founder effects, it is useful to represent our 70 populations as major regions defined by their geography and population structure. We explored genetic population structure using both microsatellites and SNP markers and various methods. Using Bayesian clustering with the CV error method (Evanno et al., 2005) for the microsatellite data, we found two primary clusters which generally split the west half of North America from the east half, with a large transition zone through New Mexico and Texas (Figure S1a). At K = 4, the four ancestries correspond generally to 1: Northern California + Central, 2: Southwest, 3: Southeast, and 4: Caribbean (Figure 1b). Southern CA has sites that are outliers (e.g., Exeter) or appear to show admixture (e.g., Mission Viejo), consistent with the complex, recent invasions known to characterize the region (Pless et al., 2020). Bayesian clustering using the SNP data reinforced the results from the microsatellites and added additional resolution in southern California, for example showing that Garden Grove and Mission Viejo each have distinct genetic signatures (Figure 3).

PCA using SNPs showed a similar pattern. The Southeast and Caribbean are grouped together on the right of the graph, along with Exeter, a known outlier that may represent a third invasion into California (Figure S2). Southern California and Southwest clustered in the top left, Northern CA was in the bottom right, and Central was in the middle. PCA using the microsatellites showed a similar pattern but with a higher level of overlap among all the populations (not shown). The consistency in the clustering results suggests the identified regions could represent major “steps” during SFE colonization and that subsequent analyses could be performed using these groupings.

We further explored the genetic structure within the Caribbean and the Southwest, since we have new populations in these regions compared to previous publications. The CV error method (Evanno et al., 2005) suggested K = 5 for the Caribbean microsatellite data (Figure S1e), but K = 3 made the most biological sense, showing moderate differentiation among the Dominican Republic (DR), United States Virgin Islands (USVI), and Puerto Rico (PR) (Figure S1d). DAPC showed a connection between some of the samples from Ocoa, DR, and PR (Figure S3). In the Southwest and Central, Bayesian clustering at K = 2 found a strong divide between Arizona and New Mexico (Figure S1b); at K = 3 western NM (through Alamagordo) separated from more eastern sites in New Mexico and Texas (Figure S1c).

### 3.2 Prediction 1: Genetic diversity decreases toward the west

The microsatellite data showed that, overall, the highest genetic diversity was found in the Southeast, followed by the Caribbean, Central, Southwest, Northern CA, and Southern CA (*R* ^2^ for longitude vs. allelic richness = 0.32, *p* < 10^−8^) (Figure 1, Table 2). After combining and standardizing the number of individuals per region, we found that Southeast and Southern California had the highest number of private alleles (alleles found in no other region), followed by the Caribbean and Central (Table 2). This is consistent with the Southeast having the oldest population and Southern California being founded multiple times from different regions. There was a stronger correlation between heterozygosity versus distance from Florida (*R* ^2^ = .53, *p* < 10^−11^) than between heterozygosity and distance from the Caribbean (*R* ^2^ = .32, *p* < 10^−11^).

**TABLE 2:** Genetic diversity of each region calculated with microsatellites

Region	Ho ± SD^a^	Ho^b^	He ± SD^a^	He^b^	AR ± SD^a^	AR^b^	Private alleles^b^
Caribbean	0.55 ± 0.032	0.56	0.56 ± 0.030	0.60	4.21 ± 0.32	6.65	4
Southeast	0.62 ± 0.035	0.62	0.62 ± 0.016	0.65	4.42 ± 0.22	6.89	8
Central	0.54 ± 0.051	0.55	0.56 ± 0.051	0.61	3.97 ± 0.44	6.31	3
Southwest	0.55 ± 0.029	0.57	0.57 ± 0.023	0.60	3.83 ± 0.018	5.23	1
Southern CA	0.47 ± 0.086	0.48	0.47 ± 0.072	0.58	2.99 ± 0.53	6.07	6
Northern CA	0.49 ± 0.015	0.49	0.52 ± 0.023	0.57	3.69 ± 0.37	4.82	0

Abbreviations: Ho, observed heterozygosity; He, expected heterozygosity; AR, allelic richness estimated by rarefaction (*n* = 30); Private alleles, number of unique alleles found only in that region.

^a^ Mean of populations within each region.

^b^ Individuals in each region combined and resampled to standardized size before analyses.

### 3.3 Prediction 2: Positive relationship between geographic and genetic distance

**FIGURE 4:**
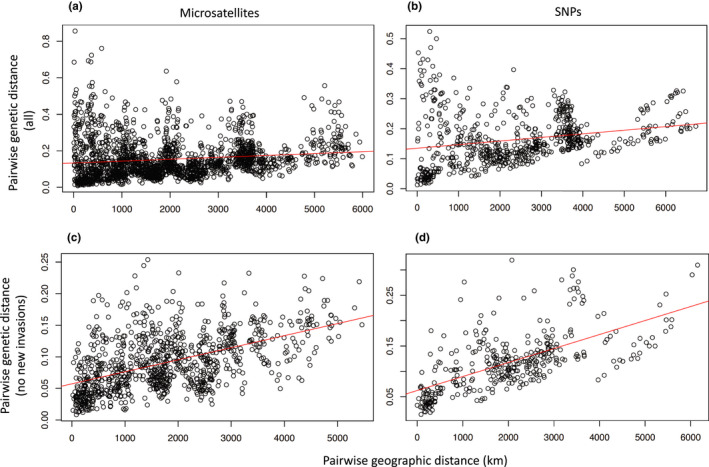
Pairwise genetic distance (linearized F_ST_) for pairs of populations as a function of the geographic distance (km) between them for (a) all microsatellite data, (b) all SNP data, (c) all microsatellite data excluding known new invasions (California, Las Vegas NV, and Albuquerque NM), and (d) all SNP data excluding known new invasions

Considering the microsatellite data, the *F_ST_* (a measure of genetic differentiation due to genetic structure) values between all population pairs (*N* = 1953) were 0.13 ± 0.070 (mean ± SD), and all values were significantly different than zero at the 0.05 level after correction for multiple tests (Table S4). Geographic distance was a significant predictor of genetic distance (calculated as $\frac{F_{\mathrm{ST}}}{\left(1‐F_{\mathrm{ST}}\right)}$) (Mantel *R* = .14, *p* = .024) (Figure 4a). After removing known new invasions from the dataset (detected in 2013 or later), the strength of the relationship increased (Mantel *R* = .52, *p* < 10^−4^) (Figure 4b). Similarly, the pairwise F_ST_ values generated from the SNP data (*N* = 630) were 0.10 ± 0.045 (mean ± SD), and all values were significantly different than zero except for the pairs between Madera and San Mateo (Table S5). Using the SNP data, there was also a significant correlation between geographic and genetic distance (Mantel *R* = 0.23, *p* = .0038) (Figure 4c), and the strength of the correlation increased when new invasions were removed (Mantel *R* = .62, *p* < 10^−4^) (Figure 4d).

**FIGURE 5:**
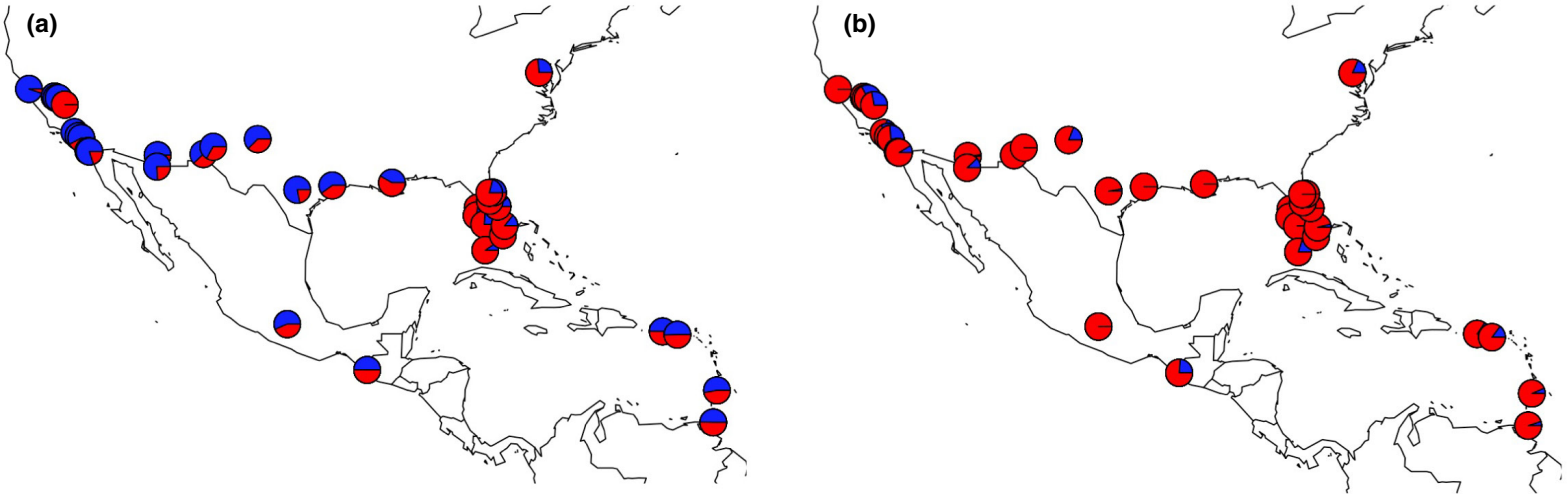
North America population structure (K = 2) generated in conStruct with the SNP dataset using (a) a non‐spatial model and (b) a spatial model that first attributes genetic variation to isolation by distance where possible

To investigate the effect of continuous processes, namely isolation by distance, on clustering, we ran conStruct for K = 1–4 using the SNP dataset. We focus on K = 2 because this model consistently produced well‐behaved MCMC posterior probability plots, and the first two layers contributed most of the variance in the majority of the runs (Table S6). The three independent non‐spatial models produced very consistent results at K = 2, showing a clear structure, especially between Florida + Exeter and California (Figure 5 and Figure S4a). After controlling for the effect of isolation by distance, the three spatial runs showed significantly less genetic structure (Figure 5 and Figure S4b), although one run continued to show Exeter as a clear outlier (Figure S4c). In all cases, the posterior probability across MCMC runs was higher for the spatial models than the non‐spatial models. The non‐spatial model showed additional structure at K = 3 and K = 4 (e.g., differences between the Caribbean and continental North America), but again the spatial model at these higher K levels showed less structure.

### 3.4 Prediction 3: Daughter groups nested within parental group

To evaluate if each putative daughter group contained a subset of the diversity in the presumed parent group, we calculated NODF for each microsatellite allele for the three regions where we expected the larger effect: Southeast, Central, and the Southwest. However, nine loci had <4 alleles, an insufficient number to detect nestedness patterns (Table 3). Of the three remaining loci, one (AG2) showed evidence of nestedness (NODF score >1SD above the neutral NODF scores), and the other two were within 1SD of the neutral model (Table 3). Additional analyses using more regions or individual populations rather than regions did not show significant nestedness.

**TABLE 3:** Test of allelic nestedness for Southeast, Central, and Southwest using microsatellite data

Microsatellite	NODF	Neutral ± SD	No. alleles total	No. alleles not fixed
AG2*	64.7	61.9 ± 1.5	20	9
AC5	49.5	49.4 ± 1.0	16	5
AC2	65.3	58.3 ± 7.8	6	4
AC1	70.8	64.5 ± 4.1	7	3
AG1	56.7	67.3 ± 3.8	7	3
B2	65.3	62.2 ± 6.8	6	3
AG5	45.2	45.2 ± 0.0	8	2
A1	41.7	51.7 ± 9.1	7	2
B3	46.2	45.2 ± 0.0	5	1
CT2	66.7	66.7 ± 0.0	3	1

Note

Asterisk (*) indicates nestedness metric (NODF) significantly greater than neutral score.

Abbreviations: Microsatellite, each microsatellite locus; NODF, nestedness metric based on the overlap and decreasing fill; Neutral ± SD, generated by randomly shuffling the rows of the nestedness matrix; No. alleles total, number of alleles for given microsatellite; No. alleles not fixed, number of alleles that vary across the three regions.

### 3.5 Prediction 4: Demographic inference modeling supports SFE

Using approximate Bayesian computation combined with supervised machine learning, we compared two invasion scenarios to test whether the data were more consistent with SFE or independent invasions from the Southeast (Figure 2). SFE had a higher posterior probability (PP > 0.922 across all four independent runs) than independent invasions from the Southeast (PP < 0.07 across all four independent runs). Under the SFE model and assuming ten generations/year, we estimated that Southern CA split from Southwest ~39–66 years ago, Southwest split from Central ~166–207 years ago, and Central split from Southeast ~322–345 years ago (Table S2). We ran the model twice using randomly selected samples from each region and twice using single populations from each region; results were similar across the four runs (Table S2).

## 4 DISCUSSION

### 4.1 Regional genetic structure

In this study, we tested whether SFE described the westward spread of *Ae*. *aegypti* across North America by evaluating the genetic diversity and population structure from a rich dataset of 70 populations. We first established relevant regions for analysis based on genetic structure; these are the hypothesized “stepping‐stone” regions for the westward spread of *Ae*. *aegypti*. We identified six genetic regions based on both microsatellites and SNPs that are consistent with previous work (Gloria‐Soria et al., 2016; Kotsakiozi et al., 2018). Broadly, we detected two primary clusters in the microsatellite dataset which split the eastern populations from the western ones with a large admixture zone through the Southwest and Central regions (Figure 2a). At higher levels of K, additional structure emerged (Figure 2), and we found genetic differentiation by geography with some outliers, specifically Exeter CA, which clusters with Florida and likely represents the third invasion into the state (Figure S1). Southern California has unusual genetic patterns (e.g., high genetic differentiation and a high number of private alleles despite low diversity), as discussed elsewhere (Pless et al., 2020). The stability of the regions we identified is indicated by older allozyme data from parts of the same distribution using collections from the 1970s and 1980s (Wallis et al., 1983). In particular, the break between the Southeast and Central around the border of Texas and Louisiana was clearly identified in these early studies.

We further explored the intra‐region structure for the Central and Caribbean, since these are the regions where we added new genetic data. Bayesian clustering using microsatellites found two primary ancestries in the Southwest and Central regions combined, clearly separating the five most eastern sites (AZ and MX) from the other sites (NM, TX, and MX) (Figure S2b). At K = 3, an additional divide emerged between Alamagordo NM and Roswell NM (Figure S2c). Despite some geographic structuring in the Caribbean (Figures S2d and S4), most analyses show high homogeneity among these sites, consistent with significant human‐mediated gene flow among the islands as previously reported (Wallis et al., 1984). Although *Ae*. *aegypti* was likely established in the Caribbean and Florida at similar times in history (1600–1700s), the lower genetic diversity and higher amount of genetic structure in the Caribbean may reflect the accumulation of genetic drift due to smaller population sizes and lower migration between the islands than between sites in Florida and/or population bottlenecks from eradication efforts on the islands (Dick et al., 2012; Sherpa et al., 2018).

### 4.2 Genetic predictions of SFE

The genetic data supported three of our predictions of SFE (#1, #2, and #4), with the Southeast as the putative source for westward spread across North America. The highest numbers of private alleles were found in Florida and the Caribbean, and genetic diversity largely decreased in populations westward (Tables 1 and 2, Figure 1). Distance to Florida was strongly correlated with heterozygosity; distance to the Caribbean was correlated to heterozygosity, but the signal was weaker. Florida likely harbors the oldest and most stable populations of *Ae*. *aegypti* in continental North America today because (1) southern Florida is hospitable year‐round for *Ae*. *aegypti* (Johnson et al., 2017), (2) vector control never fully eliminated *Ae*. *aegypti* from the region (Slosek, 1986; Soper, 1965), and (3) *Ae*. *albopictus* never fully displaced *Ae*. *aegypti* from the region (Lounibos et al., 2016).

In line with our second expectation of SFE, geographic distance was a strong predictor of genetic distance, especially after removing recent invasions on the west coast (including the highly differentiated southern California sites, and the northern California sites, which were likely founded by long‐distance human‐mediated movement) (Pless et al., 2017) (Figure 4). We also found that some of the genetic structures in North America could be explained by a continuous process like isolation by distance (Bradburd et al., 2018). Accounting for genetic variance due to isolation by distance resulted in less genetic clustering (Figure 5 and Figure S4).

Our third expectation, that daughter populations would contain a subset of the diversity contained by their parental group, could not be properly tested. The overall low genetic diversity of *Ae*. *aegypti* in the Americas, relative to that of its native Africa (Gloria‐Soria et al., 2016), resulted in a limited number of alleles per microsatellite and frequent presence of rare alleles that prevented the detection of diversity nestedness in the dataset. Exploring different regional categories and using higher sample sizes could help clarify these results. We do find support for the SFE model in the demographic analysis (prediction 4). Specifically, westward spread via a series of founder effects was better supported than independent invasions from the Southeast.

To the best of our knowledge, the SFE model has not been tested for any mosquito species, and has only been tested on a few insects more generally, see Pierce et al. (2014). We argue this model is useful in thinking about the expansion of *Ae*. *aegypti* out of Africa and across continents such as North America. Like isolation by distance, SFE can serve as a null model for *Ae*. *aegypti* genetic structure in North America, and outliers from the model warrant additional research and explanation (e.g., California being founded by multiple invasions, some from across the country). Our study also has some limitations that should be addressed in future work. While the linear decline of heterozygosity is indicative of SFE, it can also be explained by extensive admixture from an early branching lineage into later branching lineages (Pickrell & Reich, 2014). The increase in genetic distance with an increase in geographic distance can be caused by the budding pattern of migration under SFE or solely by isolation by distance (Wright, 1943). More extensive sequencing and modeling would be useful for testing these different demographic scenarios.

Clearly, *Ae*. *aegypti* can and does make stochastic long‐distance jumps, for example, from the U.S. southeast to the Netherlands (Brown et al., 2011) and to California (Gloria‐Soria et al., 2014). However, the demonstrated relevance of SFE and of environmental variables (Pless et al., 2021) on *Ae*. *aegypti* gene flow suggests short‐distance migration is important. Given the importance of short‐distance migration, controlling *Ae*. *aegypti* in one area should help protect areas around it from becoming infested. These short‐distance migrations occur across the state and country lines (e.g., the Mexico samples cluster with U.S. samples at the same longitude, not with each other), highlighting the importance of international cooperation to prevent further invasions and control vector‐borne diseases.

## AUTHOR CONTRIBUTIONS

**Evlyn Pless:** Conceptualization (equal); Data curation (lead); Formal analysis (lead); Methodology (equal); Writing – original draft (lead); Writing – review & editing (equal). **Jeffrey Powell:** Conceptualization (equal); Funding acquisition (lead); Methodology (equal); Supervision (equal); Writing – review & editing (equal). **Krystal Seger:** Resources (equal); Writing – review & editing (equal). **Brett Ellis:** Resources (equal); Writing – review & editing (supporting). **Andrea Gloria‐Soria:** Conceptualization (supporting); Data curation (supporting); Formal analysis (supporting); Methodology (equal); Supervision (equal); Writing – original draft (supporting); Writing – review & editing (equal).

## CONFLICT OF INTEREST

The authors declare no conflicts of interest.

## Supporting information

Appendix S1

## Data Availability

Genetic data (microsatellite calls and SNP array dataset) are included in the supplementary materials and are archived at Dryad (https://doi.org/10.5061/dryad.5x69p8d5j) and VectorBase (VBP0000801).
