# Supplementary material for: Evidence for serial founder events during the colonization of North America by the yellow fever mosquito, Aedes aegypti
Source: Ecol Evol. 2022 May 13;12(5):e8896. doi: 10.1002/ece3.8896 (PMC9102526; doi:10.1002/ece3.8896)
Supplement: Supplementary file 1 — Appendix S1 [file ECE3-12-e8896-s001.zip › ece38896-sup-0006-TableS2.docx]

**Table S2: DIYABC-Random Forest Analysis**

**Regional test including Southeast, Central, Southwest, and Southern California.**

**Selected scenario for parameter estimation: #1**

| **Parameter** | **Details** | **Prior** | **Posterior** |
| --- | --- | --- | --- |
| Colonization scenario | Scenario 1 – SFE moving east from SE to Central to SW to SCA | 0.5 | A) 1.000  B) 0.999 |
|  | Scenario 2 – SE is the source for each invasion | 0.5 | A) 0.000  B) 0.001 |
| Effective population size | Southeast | 10 – 100,000 | A) 60,618 [29,322 – 94,425]  B) 52,275 [24,263 – 92,450] |
|  | Central | 10 – 100,000 | A) 47,558 [17,983 – 92,121]  B) 59,208 [23,157 – 95,756] |
|  | Southwest | 10 – 100,000 | 1. A) 14,049[5,033–31,157] 2. B) 8,290[3,541–15,488] |
|  | Southern California | 10 – 100,000 | 1. 47,060[7,715–93,241] 2. 43,128[5,669–88,886] |
| Bottleneck population size | Central | 10-100,000 | A) 45,970[5,656–94,294]  B) 43,360[6,086–90,886] |
|  | Southwest | 10-100,000 | A) 51,841[8,276–96,847]  B) 47,314[4,601–94,891] |
|  | Southern California | 10-100,000 | A) 52,509[11,406–94,090]  B) 47,418[4,480–95,363] |
| Bottleneck duration | Central | 10-5000 | A) 2,561[1,542–3,572]  B) 2,445 [1,311–3,645] |
|  | Southwest | 10-5000 | A) 1,293[566–2,306]  B) 1,173[566–1,968] |
|  | Southern California | 10-5000 | A) 345[37–744]  B) 383[41–763] |
| Split time in generations | Central from Southeast | 20 – 4,000 | A) 3,306[2,312 –3,953]  B) 3,229[2,188 –3,953] |
|  | Southwest from Central | 20 – 4,000 | A) 1,825[979 –2,829]  B) 1,657[884 –2,827] |
|  | Southern California from Southwest | 10 – 1,000 | A) 640[293–938]  B) 657[290 –957] |
| Mutation Rate | Mean mutation rate | 9x10^-6^ – 1x10^-3^ | A) 9.51x10^-6^ [9.05x10^-6^ – 9.95x10^-6^]  B) 9.50x10^-6^ [9.04x10^-6^ – 9.95x10^-6^] |
| Confidence | Type II Error (simulated under scenario 1) |  | A) 0.0022  B) 0.0021 |
|  | Type I Error (simulated under scenario 2) |  | A) 0.0020  B) 0.0021 |

**Regional test including Southeast, Central, Southwest, and Southern California, using single populations (below) to represent each region**

A) Daytona Beach FL, Travis TX, Maricopa County AZ, Santa Ana CA

B) Palm Beach County FL, Alamagordo NM, Tucson AZ, San Bernardino CA

**Selected scenario for parameter estimation: #1**

| **Parameter** | **Details** | **Prior** | **Posterior** |
| --- | --- | --- | --- |
| Colonization scenario | Scenario 1 – SFE moving east from SE to Central to SW to SCA | 0.5 | A) 0.993  B) 0.922 |
|  | Scenario 2 – SE is the source for each invasion | 0.5 | A) 0.007  B) 0.078 |
| Effective population size | Southeast | 10 – 100,000 | A) 44,950 [12,223 – 88,195]  B) 61,365 [27,231 – 93,715] |
|  | Central | 10 – 100,000 | A) 44,225 [10,021 – 91,704]  B) 62,935 [29,212 – 94,140] |
|  | Southwest | 10 – 100,000 | 1. A) 23,283[3,122–77,315] 2. B) 10,108[1,695–41,830] |
|  | Southern California | 10 – 100,000 | 1. 36,153[720–94,052] 2. 28,289[842–92,623] |
| Bottleneck population size | Central | 10-100,000 | A) 48,198[6,348–95,530]  B) 52,502[7,953–96,985] |
|  | Southwest | 10-100,000 | A) 41,534[5,168–92,332]  B) 49,925[5,515–94,452] |
|  | Southern California | 10-100,000 | A) 46,429[571–96,639]  B) 37,420[1,116–94,034] |
| Bottleneck duration | Central | 10-5000 | A) 2,754[1,606–3,738]  B) 2,577 [1,404–3,633] |
|  | Southwest | 10-5000 | A) 1,445[629–2,524]  B) 1,290[651–2,227] |
|  | Southern California | 10-5000 | A) 392[45–842]  B) 466[64 –849] |
| Split time in generations | Central from Southeast | 20 – 4,000 | A) 3,454[2,566 –3,970]  B) 3,223[2,021 –3,958] |
|  | Southwest from Central | 20 – 4,000 | A) 2,066[986 –3,317]  B) 1,953[982 –2,296] |
|  | Southern California from Southwest | 10 – 1,000 | A) 687[254–978]  B) 776[419 –987] |
| Mutation Rate | Mean mutation rate | 9x10^-6^ – 1x10^-3^ | A) 9.49x10^-6^ [9.04x10^-6^ – 9.96x10^-6^]  B) 9.51x10^-6^ [9.05x10^-6^ – 9.95x10^-6^] |
| Confidence | Type II Error (simulated under scenario 1) |  | A) 0.0047  B) 0.0043 |
|  | Type I Error (simulated under scenario 2) |  | A) 0.0049  B) 0.0047 |
