# Supplementary material for: Evidence for serial founder events during the colonization of North America by the yellow fever mosquito, Aedes aegypti
Source: Ecol Evol. 2022 May 13;12(5):e8896. doi: 10.1002/ece3.8896 (PMC9102526; doi:10.1002/ece3.8896)
Supplement: Supplementary file 1 — Appendix S1 [file ECE3-12-e8896-s001.zip › ece38896-sup-0015-Legends.docx]

**Supporting information captions**

**Supporting information figures (PDF)**

**Figure S1**. Genetic structure using microsatellites and the program STRUCTURE for North America (A. K=2), the Southwest and Central (B. K= 2 and C. K=3), and the Caribbean (D. K= 3 and E. K=5). Each column represents an individual with the colors corresponding to what proportion of the individual’s ancestry comes from each of the K inferred groups.

**Figure S2.** Principal components analysis using SNPs. The colors correspond to regional colors used in Fig 1: Northern CA = pink, Southern CA = green, Southwest = light blue, Central = yellow, Southeast = red, and Caribbean = purple. The southern California population which clusters with the Southeast is Exeter, CA.

**Figure S3.** Genetic structure in Caribbean microsatellite data shown with k-means clustering (left) and discriminant analysis of principal components (right).

**Figure S4.** Results from program conStruct run with SNPs for two inferred populations (K=2). A. A relatively high amount of genetic structure was found using the non-spatial mode (all three independent runs produced results that appeared identical). As with other STRUCTURE-style plots, each column represents an individual with the colors corresponding to what proportion of the individual’s ancestry come from each of the K inferred groups. B. Very little genetic structure was found using the spatial mode, which accounts for genetic differentiation with continuous processes (i.e. isolation by distance) when possible. The second independent run of the spatial model produced identical looking results. C. An intermediate amount of genetic structure was found on the third independent run of the spatial mode.

**Supporting information tables (Excel/Word files)**

**Table S1.** Full list of populations and relevant information: Number corresponding to Figure 1 (“Number”), population name (“Population”), abbreviation (“Abbrev”), year collected (“Year”), number of individuals genotypes for microsatellites (“N_Micr”), number of individuals genotyped for SNPs (“N_SNP”), observed heterozygosity (“Ho”), expected heterozygosity (“He”), inbreeding coefficient (“F”), allelic richness (“AR”), private alleles (“PA”), latitude (“X”), longitude (“Y”), assigned region (“Region”), and whether data is being published for the first time (“New”). All genetic diversity metrics were calculated with microsatellite data.

**Table S2.** Details from DIYABC parameters and results.

**Table S3.** Nestedness results measured by NODF value across three regions (Southeast, Central, and the Southwest) and three populations (Palm Beach County FL, Ellis County TX, and Las Cruces NM). Neutral scores generated by randomly shuffling the rows of the nestedness matrix.

**Table S4.** Pairwise F_ST_ values and p-values using microsatellite data.

**Table S5.** Pairwise F_ST_ values and p-values using SNP data.

**Table S6**. Layer contribution to variance in spatial vs non-spatial models for conStruct run on SNP data.

**Supporting information datasets**

**Dataset S1.** Microsatellite data in Genepop format (text file)

**Dataset S2.** SNP data in Plink format (bed/bim/fam files)
