# Supplementary figures and images for: Evidence for serial founder events during the colonization of North America by the yellow fever mosquito, Aedes aegypti
Source: Ecol Evol. 2022 May 13;12(5):e8896. doi: 10.1002/ece3.8896 (PMC9102526; doi:10.1002/ece3.8896)

N. CA      S. CA      Southwest      Central      Southeast      Caribbean

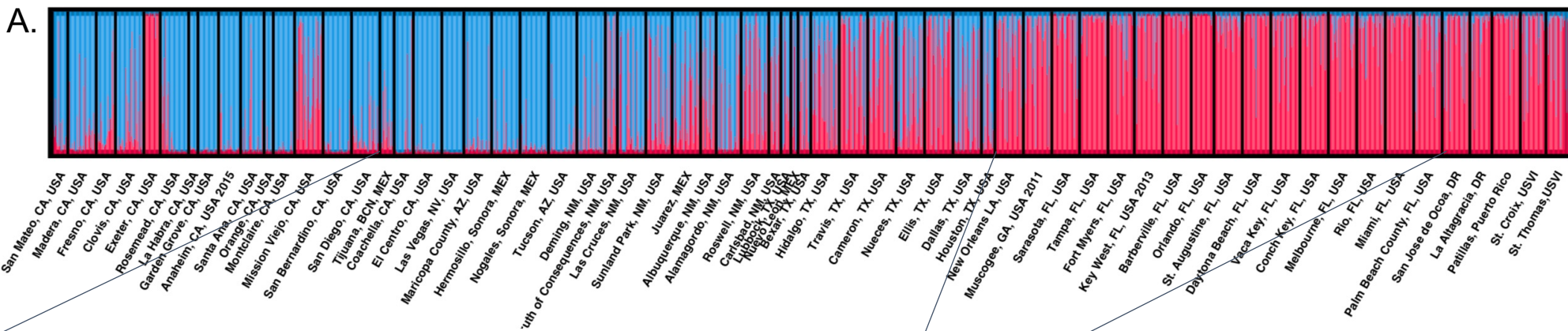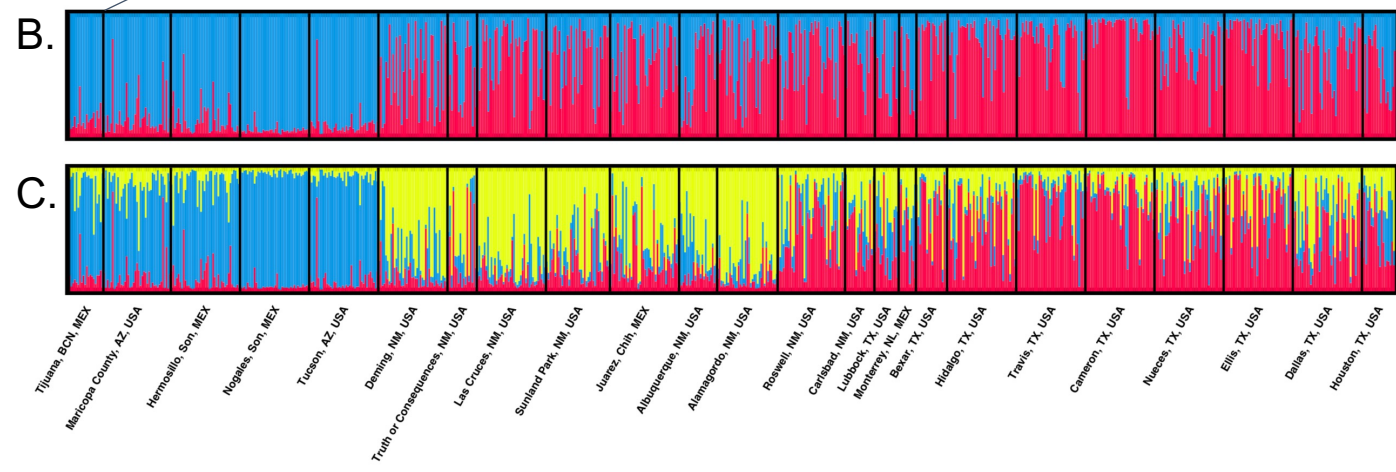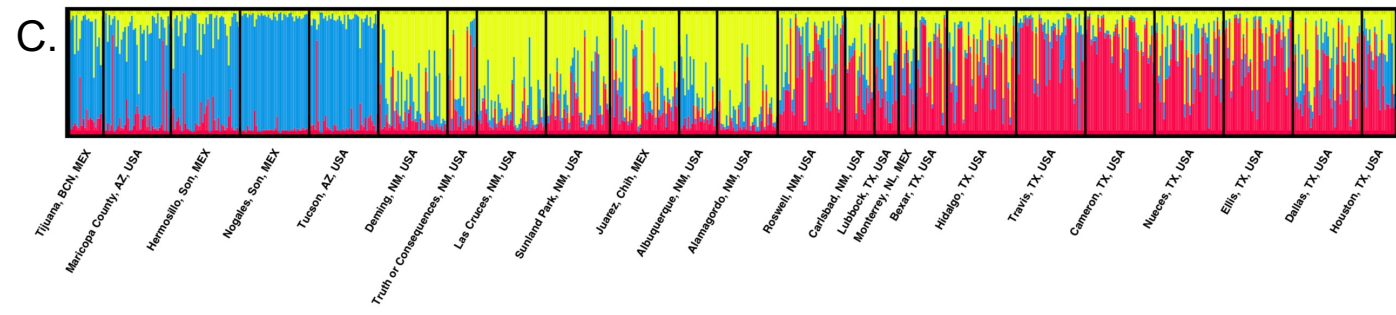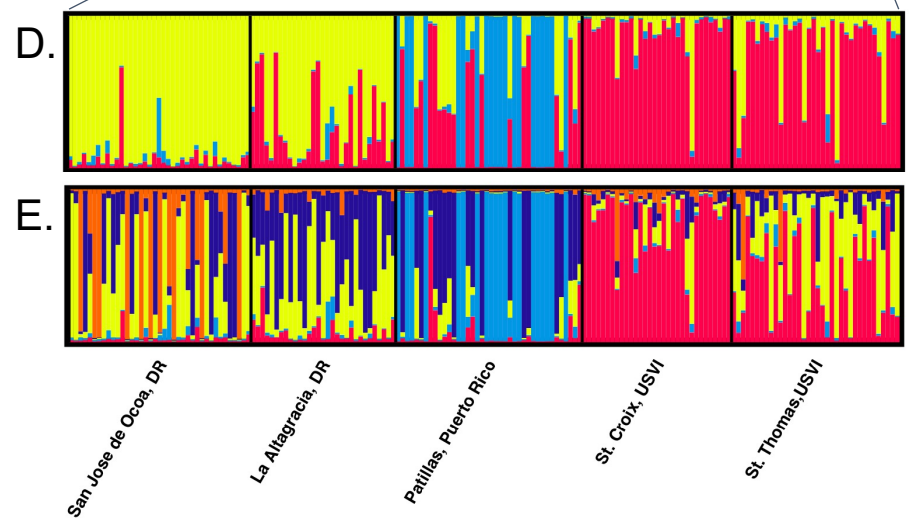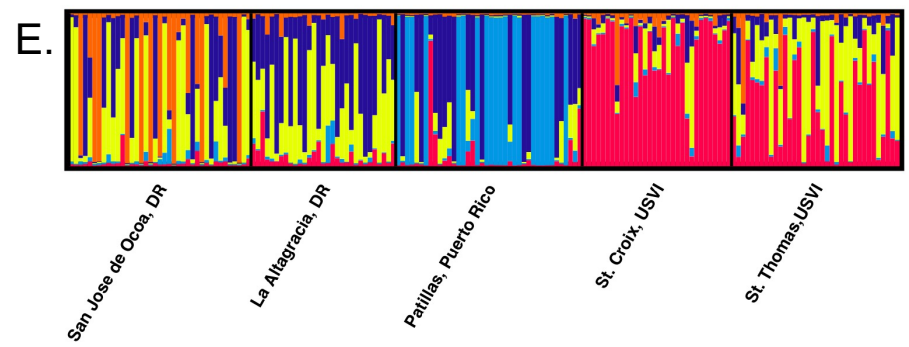

Supplement: Supplementary file 1 — Appendix S1 [file ECE3-12-e8896-s001.zip › ece38896-sup-0001-FigS1.pdf]

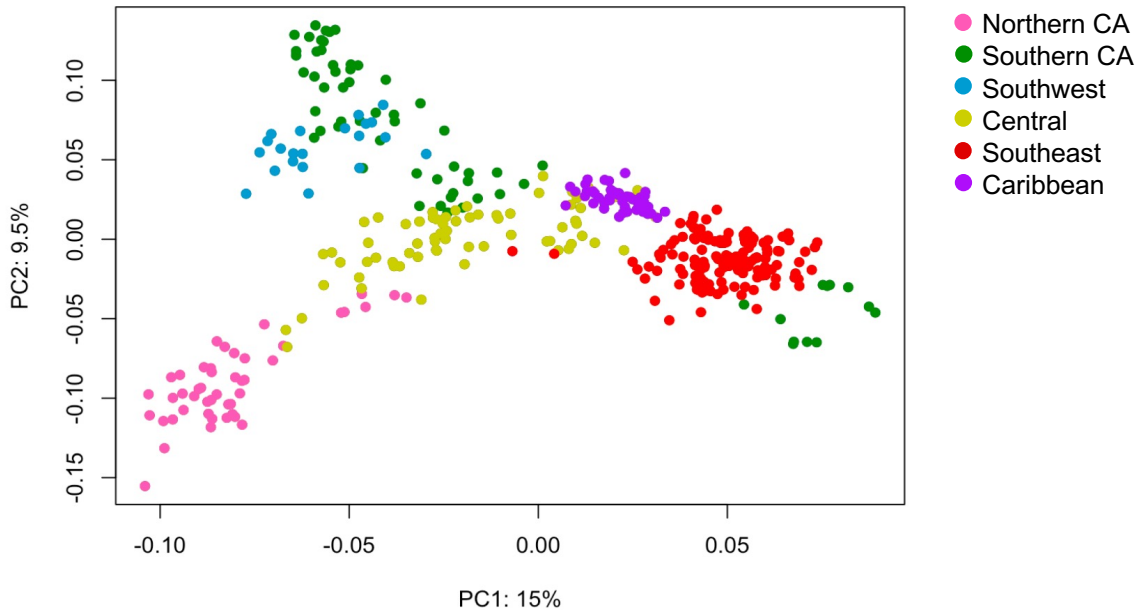

Supplement: Supplementary file 1 — Appendix S1 [file ECE3-12-e8896-s001.zip › ece38896-sup-0002-FigS2.pdf]

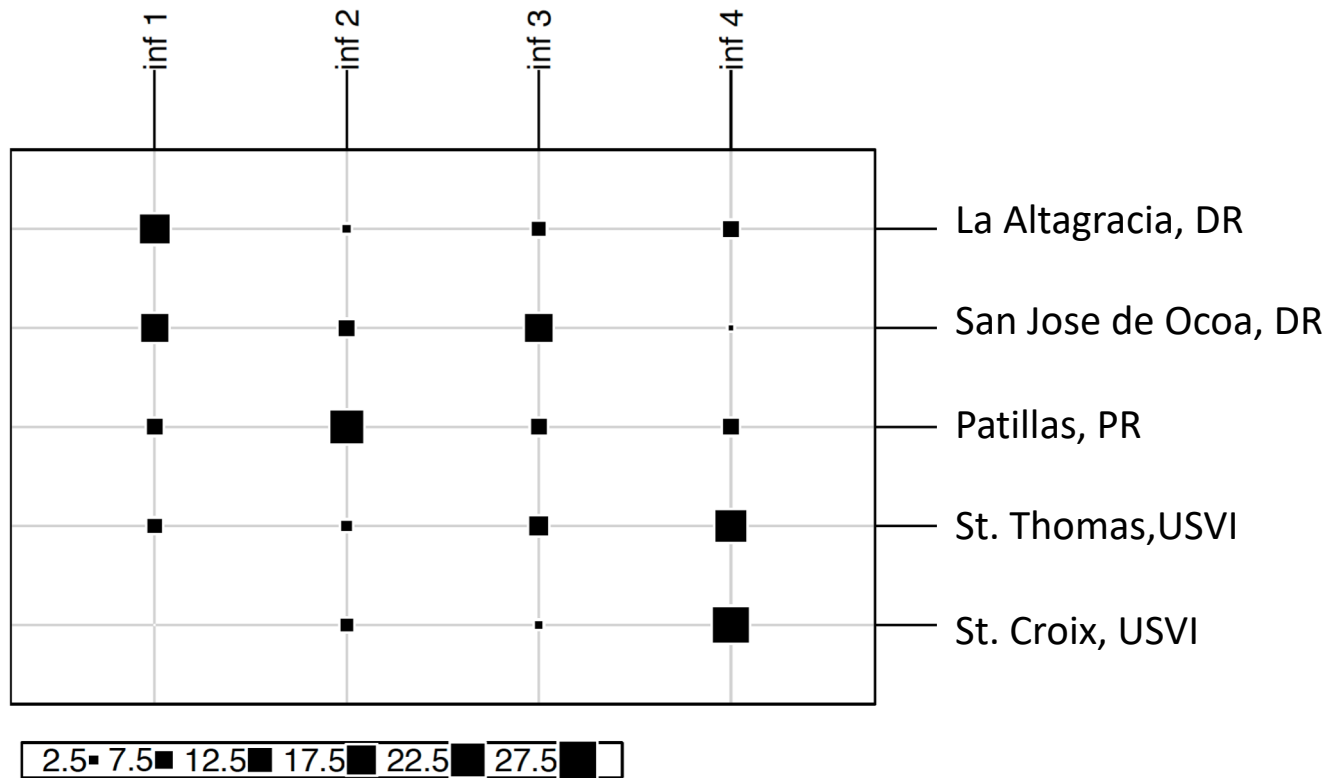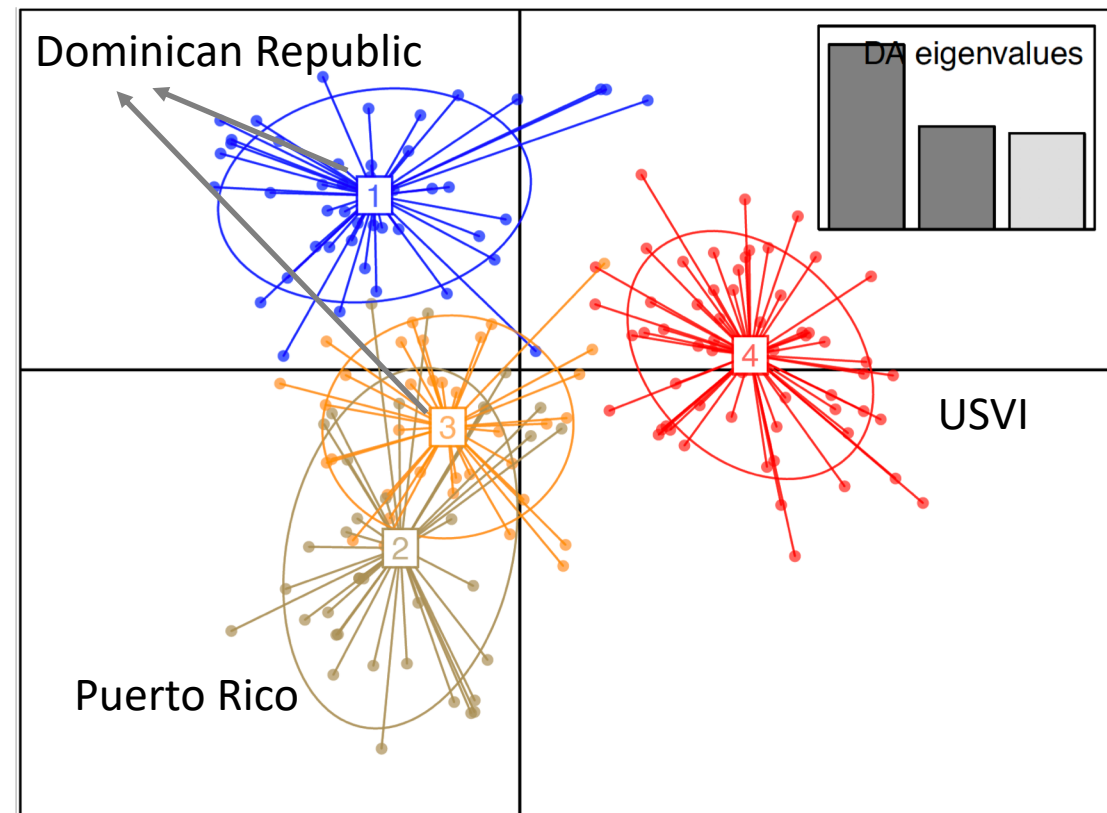

Supplement: Supplementary file 1 — Appendix S1 [file ECE3-12-e8896-s001.zip › ece38896-sup-0003-FigS3.pdf]
