# Supplementary material for: Evidence for serial founder events during the colonization of North America by the yellow fever mosquito, Aedes aegypti
Source: Ecol Evol. 2022 May 13;12(5):e8896. doi: 10.1002/ece3.8896 (PMC9102526; doi:10.1002/ece3.8896)
Supplement: Supplementary file 1 — Appendix S1 [file ece38896-sup-0004-FigS4.pdf › ece38896-sup-0004-FigS4.pdf]

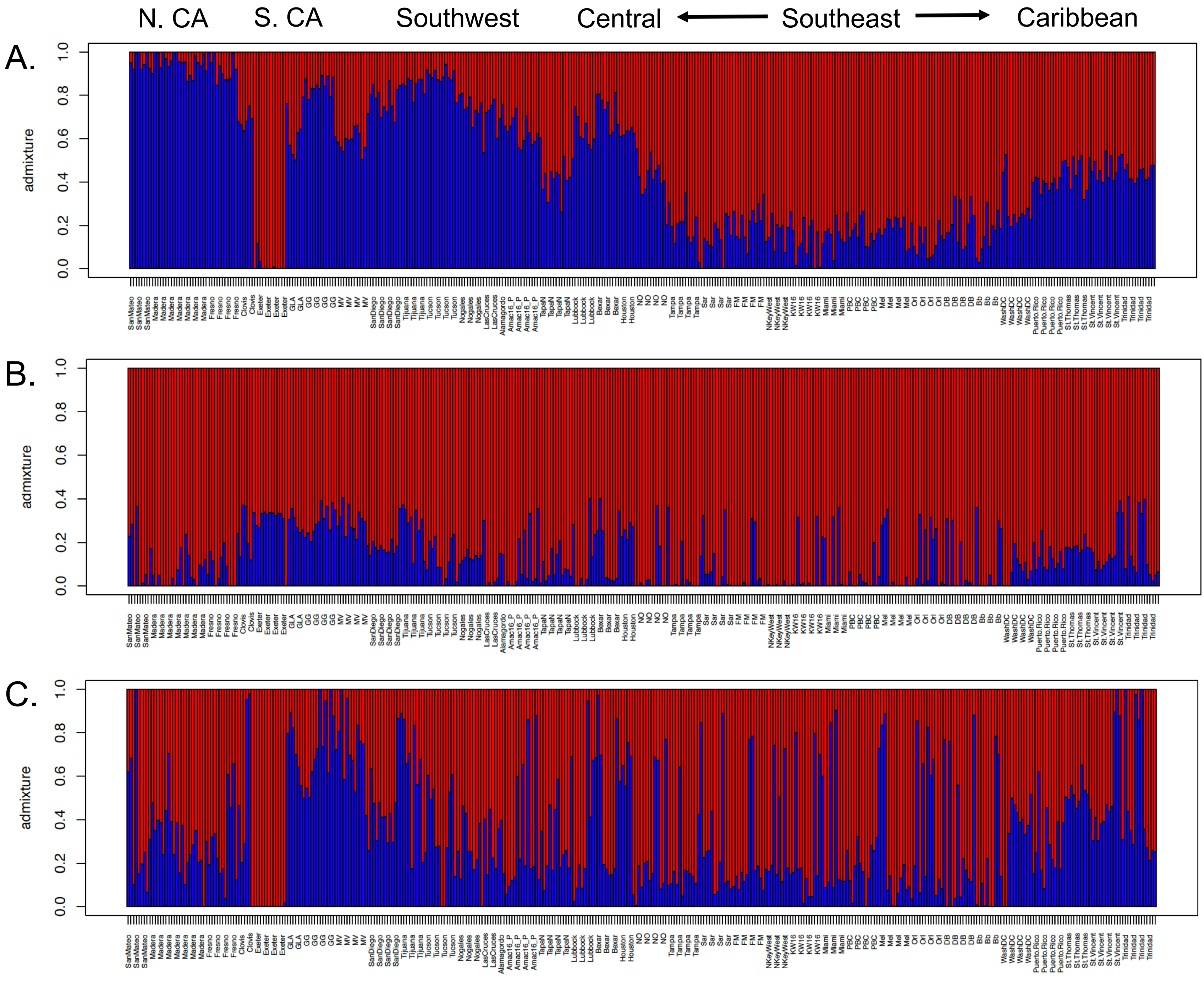
N. CA
S. CA
Southwest
Central
Southeast
Caribbean
A.
B.
C.
admixture
0.0
0.2
0.4
0.6
0.8
1.0
SanMateo
Madera
Fresno
Clovis
Exeter
GLA
GG
MV
SanDiego
Tijuana
Tucson
Nogales
LasCruces
Alamagordo
Amac16_P
TapaN
Lubbock
Bexar
Houston
NO
Tampa
Sar
FM
NKeyWest
KW16
Miami
PBC
Mel
Orl
DB
Bb
WashDC
Puerto.Rico
St.Thomas
St.Vincent
Trinidad
